# Co-evolution of conditional cooperation and social norm

**DOI:** 10.1038/s41598-023-43918-w

**Published:** 2023-10-03

**Authors:** Balaraju Battu

**Affiliations:** Science Division, New York University Abu Dhabi, Abu Dhabi, United Arab Emirates

**Keywords:** Computational biology and bioinformatics, Evolution, Physics

## Abstract

The co-evolution of conditional cooperation and social norms has garnered significant attention, yet the underlying mechanisms remain elusive. Social norms result from empirical expectations, individual expectations of group behavior, and normative expectations, the population's expectations of individual behavior. Aligning these expectations aids in norm formation, but diverse individual reactions to observed behavior and their sensitivity to norm conformity can be challenging. In our study, the agents are initially endowed with diverse conditional expectations, which mirror their anticipations regarding group behavior and their inherent inclination to conform to social norms, indicative of their sensitivity to psychic costs. These agents engage in a repeated public goods game, where their decisions to cooperate are shaped by their conditional expectations and the observed levels of cooperation within their group. Concurrently, free riders experience psychic costs determined by the overall level of cooperation, contribution costs, and the individual’s inclination to adhere to social norms. Remarkably, our simulations unveil that agents commencing with random conditional expectations and a propensity to conform to norms can adapt to lower conditional expectations and moderate their propensity to conform to norms when initial cooperation levels are high and the contribution cost is reduced. Interestingly, increasing contribution costs intensify the population’s response to norm enforcement, but this doesn’t always result in a corresponding increase in cooperation. By incorporating population diversity and accounting for empirical and normative expectations within our model, we gain valuable insights into the intricate relationship between conditional cooperation and the emergence of social norms.

## Introduction

Cooperation means working together for a common benefit^1–3^, in which a cooperative individual incurs personal costs to benefit another or social group. Sometimes, cooperation presents social dilemmas, situations like groups hunting, paying taxes, and joining the armed forces. Understanding how individuals overcome self-interest and engage in positive reciprocity has been a long-standing problem in social dilemma, which arises in collective action problems^1,4–8^ . In these circumstances, there are consistent behavioral patterns observed in repeated public good games across societies^2,9,10^. These patterns illustrate individuals’ inclination toward conditional cooperation, where their cooperative behavior depends on past levels of cooperation, and they respond with negative reciprocity when confronted with instances of free-riding within their social groups. In these conditions, individuals are often willing to punish the free riders even at personal costs^11^ . Surprisingly, in a few societies, individuals engage in antisocial punishments in response to altruistic punishments^9,12,13^. In these societies, cooperation does not emerge, even when altruistic punishments exist. These differences can be attributed to variations in upholding social norms^9,14,15^. Establishing norms in a population of heterogeneous individuals poses a challenge because individuals exhibit a range of responses to the observed behaviors of others and react to the sanctions differently. To establish cooperation successfully, individuals must adjust their conditional behavior in response to observed actions and become receptive to sanctions. This co-evolution of conditional behavior and sensitivity to norms is pivotal in fostering cooperation.

Whenever a human society forms groups, it forms social norms^16–21^. Individuals judge the actions of others, i.e., approve of certain behaviors and disapprove of others. It is well recognized that social norms provide solutions among genetically unrelated individuals in collective action problems^3,20–24^, such as addressing climate change ^25^, promoting vaccination ^26^, and implementing social distancing measures during the COVID-19 pandemic ^27–29^. According to Bicchieri^21,24^, a social norm represents a conditional preference that relies on both empirical and normative expectations. Empirical expectations refer to the commonly observed behavior within the social group, and normative expectations arise from behavior widely accepted by most of the population^30^. On the other hand, within the population, if the majority engages in free riding, an individual may perceive free riding as a typical behavior and feel less compelled to cooperate^24^. Further, the group members are less compelled to sanction the norm violators. To exemplify this, consider wearing a mask or social distancing during the COVID-19 pandemic^28^. When an individual recognizes the widespread adoption of mask-wearing in a community and witnesses consequences or social disapproval directed at those who opt not to wear masks, wearing a mask transforms into a prevailing social norm. Recently, various complex scenarios have been considered to understand social behavior in the presence of norms^2,22,24,27,31–43^. These approaches include bottom-up ^19,28,43–45^, i.e., the emergence of norms through repeated interaction, and top-down, i.e., norms are prescribed, such as the intervention of law^34,46^, religion^47^, institutions^48,49^, and cultural leaders^50^.

Other than social norms, in the literature, two significant approaches have emerged to address the challenges of cooperation without institutional arrangements. The first approach assumes that individuals are rational and driven by self-interest; cooperation is elucidated by considering the long-term consequences of present actions^48,51–53^ and their reputation^54–56^. In contrast, the second approach posits that individuals engage in reciprocity and retaliation towards those who exploit cooperation, even at high personal costs (Fehr and Schurtenberger^2,57^).

In collective scenarios^4,8,10,40,58–60^, such as social dilemmas^5,6,8^, the second approach emphasizes the inherent altruistic nature of human behavior ^61–71^, explicitly highlighting concepts such as reciprocity, fairness, and retaliation^72^. Several significant models rooted in reciprocity have been proposed elsewhere^73^. Furthermore, physics-based models have also yielded valuable insights into the development of cooperation^74^. For instance, population diversity alone could be a condition to promote cooperation^75,76^.

To deepen our understanding of the evolution of cooperation, researchers have explored the co-evolution of individuals’ strategies and their inherent traits and gene-cultural evolution^77,78^. Regarding cooperative interactions among kin, organisms must coevolve to identify their kin and to decide whether to extend assistance or withhold it^79^. These same abilities are relevant when examining human interactions (Riolo et al.^80^), establishing connections with whom to engage^81,82^, and whom to educate^83^ are pivotal considerations. It’s important to acknowledge that not all individuals have equal opportunities for interaction^84^, and an individual’s spatial position can influence their status, thus shaping strategic interactions and the evolution of cooperation^76^. A comprehensive review of co-evolution, encompassing spatial structure and heterogeneity models, can be found elsewhere^85^. These studies have inspired the study of coevolutionary dynamics of individual traits and strategies and extended to public goods games by considering group size and participant numbers(Santos et al.^75^).

In the realm of the conditional nature of cooperation, theoretical examinations^68,86–89^ and experimental investigations have explored conditional cooperation^11,90–93^. The author of this paper has undertaken a comprehensive analysis of the evolution of conditional cooperation, considering various scenarios, for example, by scrutinizing a wide array of imitation strategies (Battu^71^; Battu et al.^70^; Battu and Rahwan^68^; Battu and Srinivasan^69^). In this context, individuals tend to emulate those who not only achieve higher payoffs but also enjoy a superior reputation. One avenue of exploration centers on the application of generous penalties, which are directed towards unconditional free riders—individuals who persistently engage in free-riding regardless of the actions of others. Additionally, penalties are administered probabilistically against negative reciprocators, those individuals who resort to free-riding only in response to its prevalence within the population. Recent research has further demonstrated that many resolute altruists bolster conditional cooperation(Battu and Rahwan^68^). These altruists influence positive reciprocity and play a pivotal role in shaping social learning. However, it’s important to highlight that these studies primarily focused on empirical expectations and did not delve into normative expectations and individual’s heterogeneous responses to sanctions.

## Method

Two pivotal evolutionary models are widely considered for comprehending the evolution of social norms. The first model delves into norm internalization^45^, while the second primarily concentrates on how social structure influences norm enforcement^19^. The former model is grounded in the idea of altruistic punishments, and individuals maximize their utility function to internalize norms. In contrast, the latter model fails to distinguish between social norms and conventions. Both models focus on individual behaviors and associated penalties. Yet, they do not consider empirical and normative expectations, which are the hallmarks of social norms. Along these, it is supposed that indirect reciprocity helps to understand the evolution of moral norms by considering the receiver’s reputation^56,94,95^. They define moral norms based on two factors, such as an assessment rule and an action rule. Moral norms are deeply connected with the judgment of the reputation of individuals, in terms of some actions being good and some being bad.

Nonetheless, it is essential to note that social norms diverge from moral norms and are contingent upon the conditional expectations held by the population, which hold significant relevance for individuals within their respective social groups.

In contrast to earlier models, the proposed model takes into account both empirical and normative expectations when defining social norms and the diversity of individuals in their responses to these expectations. The modeling approach considers previous advances made in defining norms^21^ and co-evolutionary models^85^. The proposed model draws inspiration from the pioneering work of Bicchieri ^24,30^. According to Bicchieri, the social norm is a conditional preference that depends on empirical and normative expectations. Empirical expectations revolve around an individual’s beliefs regarding the behavior they anticipate from others. In comparison, normative expectations pertain to an individual’s convictions concerning what others deem as socially acceptable behavior for them. We model the agents and sanctions in the following manner:

(*a*) the agents are conditional cooperators; hence, their behavior depends on the empirical expectations, i.e., their assessment of cooperation levels in previous rounds expectations are represented as the disapproval of cooperators within the social group towards free-riding behavior. It is important to note that normative expectations impose psychological costs on agents, and all individuals may not respond to the sanctions similarly.

(*b*) violating a norm doesn’t necessarily result in social sanctions unless most of the reference group conforms^24^. Norm enforcement is distinct from punishments and rewards in dyadic interactions in which individuals are penalized or rewarded by their counterparts. The power to enforce norms comes from the individual concern for fairness. Norm enforcement depends not only on the actions of an individual, say free riding, but also on how many individuals in their social group conformed or cooperated. A few people cooperating signals acceptance of free riding or norm violation, and the individuals in their social group are less likely to sanction the norm violators^28^.

We consider a population of heterogeneous agents whose cooperative choices are contingent upon the previous cooperation levels. The individuals who refrain from contributing experience psychic costs, which are influenced by factors such as contribution cost, levels of cooperation, and the agents’ sensitivity to psychic costs. Together, these parameters contribute to understanding social norms and conditional cooperation in collective action scenarios. We present the formal model below.

### Evolutionary dynamics

The model employs an evolutionary framework where agents adapt and update their strategies based on their performance and the strategies of other agents in the population (Axelrod and Hamilton^1^; Battu and Rahwan^68^). Agents with successful strategies are more likely to be replicated and influence the overall population dynamics.

In the envisioned model, agents participate in a series of iterative public goods games (PGG). Within each cycle of this game, individuals commence with an equivalent initial endowment. They then face a voluntary decision: either refrain from contributing or commit their complete endowment to a shared pool. After consolidating all contributions, the amassed pool undergoes augmentation by a multiplier exceeding one, and it is subsequently shared evenly among all participants, regardless of their contributions to the pooled resources. The conditional preferences of agents modeled based on behavioral trends observed in the repeated PGG: (i) an initial prevalence of cooperation among most individuals and (ii) a subsequent reduction in cooperation over successive rounds. The hypothesis posits that a significant portion of the population adopts the role of conditional cooperators while individuals display heterogeneity in their conditional behaviors. To illustrate, considering previous levels of cooperation, not all individuals exhibit uniform behavior; specific individuals might cooperate while others might abstain. The model assumes individuals exhibit behavior similar to conditional cooperators, showcasing a propensity to respond conditionally based on others’ actions, and underscores the existence of diversity in the population’s conditional tendencies. The population composition and the strategies of conditional cooperation have been adopted from a prior publication by the author^68^. The formal model follows below.

### Population

The model comprises agents with diverse characteristics, such as the agents can vary in their conditional nature and propensity to hold social norms.

Each individual within the population possesses two inherent characteristics upon birth: an arbitrary Conditional Cooperative Criteria (*CCC*) and a disposition to adhere to conditional cooperative norms (*γ*). The *CCC* serves as an expected past cooperation level within the group, shaping their willingness to contribute to the communal pool or public good. For instance, let’s take an agent denoted as *i* with a *CCC* value of *m*. When this agent participates in a repeated public goods game during the *r*th round, their inclination to contribute to the public good increases if *m* is less than *nC*_(*r-1*)_ Here, *m* represents the predicted past cooperation level—precisely, the count of cooperative agents in the previous round—while *nC*_(*r-1*)_ stands for the cooperation level in the preceding (*r-1*)th round, i.e., empirical expectations^21,24,96^. Agents with *m* = *0* exhibit altruistic tendencies, as their contributions are unaffected by past cooperation. Conversely, when *m* = *N* (assuming *N* is the population size), agents emulate unconditional free riders. Agents with values of *0* < *m* < *N* exemplify the traits of conditional cooperators, demonstrating a concern for the past cooperation level as a basis for contributing to the public good. We also consider a noise parameter *β,* which modulates the cooperative decision of the individuals when the individual does not have access to the perfect information about past cooperation levels. For more comprehensive information regarding the parameter *β*, please refer to Eq. (1).

The parameter *γ* is modeled considering the following psychological factors*:* People generally abide by social norms to evade possible tangible (financial losses such as fines) or psychological disadvantages (aversion to guilt^2^). All the agents may not experience financial or psychological losses similarly. In the model, an agent with *γ* = 0 shows no sensitivity to psychological costs, whereas an agent with *γ* = 1 is susceptible to such costs. This could be the result of the degree of socialization of individuals; oversocialized individuals are susceptible to psychic costs compared to less socialized individuals^45^.

### Conditional cooperative strategies

All the agents play a repeated PGG, and the agents are aware of the past cooperation level and the agents’ *CCC* values. In a typical PGG, the agent’s decision to contribute to the PGG is based on whether the observed cooperation level in the previous round exceeds its *CCC*_*j*_ value. Specifically, in the *r*^*th*^ round, the agent *j* determines their contribution to the public good using the following probability equation^68^:

1 $$p_{j} = {1}/\left( {{1} + {\text{exp}}\left( { - \beta \left( {nC_{{({\text{r}} - {1})}} - CCC_{j} } \right)} \right)} \right)$$

Here, *nC*_(r-1)_ represents the cooperation level observed in the (*r-1*)th round. The parameter *β* is an exogenous factor influencing the uncertainty associated with accessing social information about the previous round’s cooperation level. As *β* increases towards infinity, the noise level decreases, indicating that agent *j* has perfect information about *nC*_(r-1)_. In this scenario, the agent’s decision is solely based on their conditional rule (*nC*_(r-1)_–*CCC*_*j*_). On the other hand, when *β* is 0, all information is lost, implying that the agent cannot access any information about *nC*_(r-1)_. Furthermore, when the difference between the cooperation level in the previous round *nC*_(r-1)_ and the agent’s *CCC*_*j*_ equals 0, it signifies that the agent is uncertain or confused about whether to contribute. In such cases, when *β* approaches 0 or (*nC*_(r-1)_– *CCC*_*j*_) equals 0, the agent’s contribution to the PGG is determined by a coin toss.

For values of *β* within the range 0 < (*nC*_(r-1)_–*CCC*_*j*_)*β* < 1, the agent’s contribution to the public good follows a probability range of 0.5 < *p*_*j*_ < (*e*/(*e* + 1)). This implies that the likelihood of an agent contributing to the public good falls between 0.5 and the ratio of Euler’s number (*e*) divided by (*e* + 1), given the same level of cooperation observed in the previous round. Furthermore, based on these probabilities, we can deduce that agents with higher *CCC*_*j*_ values are less likely to contribute than those with lower values, assuming the cooperation level remains constant. In a population of size *N*, an agent with a *CCC*_*j*_ value equal to *N* behaves as an unconditional free rider. This means that regardless of the cooperation level in the previous round, the agent will not contribute to the public good. On the other hand, an agent with a *CCC*_*j*_ value of 0 behaves as an unconditional cooperator, indicating that they will contribute to the public good regardless of the previous round’s cooperation level.

Agents accumulate their payoff from the provision of the public good, which is given by:

2 $$\pi_{{{\text{pgg}}i}} = \left( {U - c_{i} } \right) + \left( {h/N} \right)\sum \left( {i = {\text{1 to N}}} \right)c_{i}$$

In this equation, U represents the initial endowment, *c*_*i*_ is the contribution cost, *h(*> *1)* is an enhancing factor of the collected good, and *N* is the population size.

### Enforcement of social norm

Once the agents have made their contribution decisions in the *r*th round, the free-riding agents potentially experience psychic costs. These costs serve as a deterrent to discourage such behavior. These costs can arise from guilt aversion^97,98^ or the fear of reputational consequences^99–104^ cognitive dissonance^105^, which arises when an individual’s actions conflict with the established norms of society. These costs are determined by the number of cooperative agents (*nC*_*r*_), the cost of donations (*c*_*i*_), and the agent’s *γ*_*i*_ value. Mathematically, a free-riding agent *i* experiences a psychic cost (*π*_*psyi*_) equal to the product of *nC*_*r*_, *c*, and *γ*_*i*_:

3 $$\pi_{{{\text{psy}}i}} = nC_{r} \times c_{i} \times \gamma_{i}$$

The norm violators experience a higher psychological cost when more agents cooperate and when the contribution costs are higher. The level of psychic cost experienced by an agent depends on their *γ* value. A free-riding agent with *γ*_*i*_ = 0 does not experience any psychic cost, while the same agent with 0 < *γ*_*i*_ < 1 experiences a moderate level of psychic cost. On the other hand, with *γ*_*i*_ = 1 the agent experiences a higher psychic cost. The cumulative payoff of an agent after a typical round is given by:

4 $$\pi_{{\text{j}}} = (\pi_{{{\text{pgg}}i}} - \pi_{{{\text{psy}}i}} )$$

#### Population updating

In evolutionary game theory, “payoffs” is often used interchangeably with “fitness.” To update the population strategies, we employ a pairwise comparison process^7^. This process involves randomly selecting and matching two agents, and a higher-fitness agent has a probability of replacing a lower-fitness agent. Let’s consider the scenario where agent *i* is matched with agent *j*. The probability of agent *i* imitating the strategy of agent *j*, including their *CCC*_*j*_ and *γ*_*j*_ values, is given by:

5 $$p_{ij} = \pi_{j} /\left( {\pi_{j} + \pi_{i} } \right)$$

Here, *π*_j_ represents the fitness (payoff) of agent *j*, while *π*_*i*_ represents the fitness of agent *i*. The probability p_ij_ signifies the likelihood that the lower-fitness agent *i* will imitate the higher-fitness agent *j*. In other words, the probability of agent *i* adopting the strategy, *CCC*_j_, and *γ*_*j*_ of agent *j* is determined by comparing their relative fitness. The higher the fitness of agent *j* relative to agent *i*, the greater the probability that agent *i* will imitate the agent *j*’s strategy. During the selection process, a mutation rate of 5% is introduced, meaning that each agent has a 0.05 probability of mutating. Mutations occur by adding a random value drawn from a Gaussian distribution with a mean of 0 and a standard deviation 5 to the updated *CCC*_*j*_ value. The mutation process aims to introduce variability in the population by modifying the *CCC*_*j*_ values of individual agents. The random value drawn from the Gaussian distribution represents the magnitude and direction of the mutation. In the case of mutation of *γ,* with 0.05 probability, replace updated *γ* with a randomly drawn value from a uniform distribution [0, 1]*.*

To ensure the mutated *CCC*_*j*_ values remain within a valid range, several constraints are applied:

- Maximum Mutation Value: The maximum mutation value is *N*/2, where *N* represents the population size. This limit prevents excessively large mutations that could disrupt the dynamics of the population.
- Minimum Mutation Value: The minimum mutation value is set at -*N*/2. This limit prevents mutations that could result in negative *CCC*_*j*_ values.
- Rounding Off Positive Exceeding Mutations: If the mutated *CCC*_*j*_ value exceeds the population size *N*, it is rounded off to *N*. This ensures the *CCC*_*j*_ values remain within the valid range of 0 to *N*.
- Rounding Off Negative Mutations: If the mutated *CCC*_*j*_ value is negative, it is rounded to 0. This ensures that negative *CCC*_*j*_ values, which are not meaningful in this context, are not considered.

These steps, including mutation, constraint application, and rounding, are repeated for a specified number of generations to simulate the evolution of the population over time. The mutation process introduces random variations while the constraints maintain the validity and coherence of the *CCC*_*j*_ values within the population.

## Simulations

### Parameter settings

The model considers various parameter settings, such as the cost of contribution and the initial cooperation levels, which play a crucial role in shaping the co-evolution of social norms and conditional cooperation. These parameters determine the trade-off between individual costs and collective benefits and the starting point for the emergence of norms. By simulating agents’ interactions and strategic adaptations within this agent-based model, we explore how different parameter settings and agent characteristics influence the establishment of conditional cooperative norms and cooperation. The co-evolution of conditional cooperation and norms takes place when agents’ conditional thresholds (*CCC*) are lower than the past level of cooperation, and agents become responsive to social sanctions (*γ* > 0). The model provides insights into the conditions that facilitate or hinder the co-evolution of social norms and conditional cooperation, shedding light on the dynamics of cooperation in social systems.

In the simulations, the following parameters and processes are applied:

- *Population Size* The population size is set to *N* = 100, meaning there are 100 agents.
- *Initialization of* *CCC*_*j*_ *and* *γ*_*j*_ Each agent’s *CCC*_*j*_ value is randomly drawn from a uniform distribution within the range [0, *N*]. This assigns a random initial expected cooperation level to each agent. The *γ*_*j*_ value is also drawn from a uniform distribution within the range [0, 1], representing the initial propensity to hold conditional cooperative norms.
- *Public Goods Game (PGG)* All agents participate in a repeated PGG with an initial endowment U = 10 and a contribution cost *c* units. Each agent determines their contribution to the PGG using Eq. (1), which involves comparing the observed cooperation level in the previous round with their *CCC*_*j*_ value.
- *Strategy Updating* After each generation of the PGG, all agents update their strategy according to Eq. (5). An agent’s cumulative payoff (*π*_*j*_) is based on the provision of the public good and any incurred psychic costs (see Eq. (4)). The strategy updating process determines the relative fitness of each agent, which influences their probability of being imitated by other agents in the population.

### The rationale for the experimental conditions

Within the model, the initial level of cooperation impacts both empirical expectations and norm enforcement. The contribution cost directly affects the magnitude of the attainable public good, while noise affects decisions related to conditional cooperation. The outcomes may manifest in the evolutionary dynamics of cooperation, distribution of *CCC*, and *γ*. By following the above steps and processes, the simulation captures the dynamics of the population, including the evolution of *CCC*_*j*_ values, *γ*_*j*_ values, and strategies over multiple generations. In the simulations, the following experimental conditions and outcome measures were considered:

### Experimental conditions

Population Size (*N*) = 100. Enhancing Factor (*h*) = 1.5. Initial Endowment (*U*) = 10. Percentage Cooperation levels (α): Varied from 0 to 50% in increments of 10%. Cost of contribution (*c*_*i*_): {0.1,0.3,0.5,0.7,0.9,1}. Noise Level (*β*): Varied from 0 to ∞, with specific values ranging from 0 to 2 and ∞. Number of Generations (*g*_*n*_) = 20,000. Each experimental condition was repeated 30 times.

### Outcome measures

(*i*) Evolution of cooperation levels, *CCC* values, and *γ* across Generations: The cooperation level in each generation was measured by calculating the percentage of agents who contributed to the public goods game. The evolution of *CCC* and *γ* was tracked across the generations. (ii) Asymptotic behavior of {cooperation, *CCC*, and *γ* }: represents the average values over the last 5000 generations out of 20,000. (iii) The distribution of γ and *CCC* values offers valuable insights into the dynamics of social norms. The MATLAB code was used to conduct the simulations and produce the results. Please refer to the Supplementary Material(SM)for further details and the MATLAB code used in the simulations.

## Results

The simulations reveal that lower contribution costs and high initial cooperation in public goods games lead to the population adapting to lower *CCC* values and moderate γ values. These adaptations establish high levels of stable cooperation. Cooperation is not established when the population starts with a lower level of cooperation and high contribution costs. The parameter *β* modulates the dynamics but does not destroy the establishment of cooperation unless the value is close to zero. The specific results, including statistical measures and graphical representations, are detailed below.

**Figure1:**
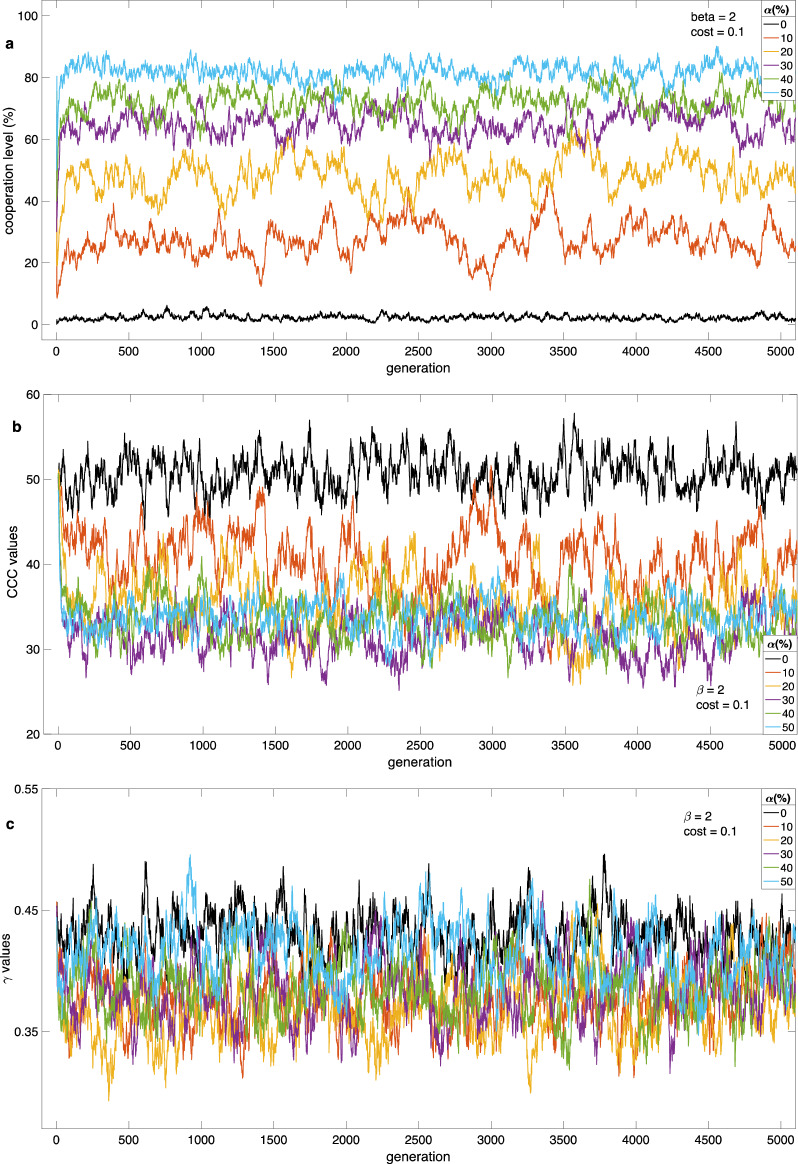
Population dynamics over the initial 5000 generations. (**a**) We observe the evolution of cooperation levels. The results indicate that when the initial cooperation level increases, with a contribution cost of 0.1, the population is capable of establishing a higher degree of stable cooperation. (**b**) Illustrates the evolution of *CCC* values. The findings reveal that as the initial cooperation increases, the population adapts to lower *CCC* values. (**c**) Showcases the evolution of *γ*. The population adapts to lower γ values when the initial cooperation is lower than when the initial cooperation level is high.

Figure 1 illustrates the graphical depiction of various parameters in population dynamics throughout the initial 5000 generations. In this examination, we obtained initial *CCC* values from a uniform distribution spanning [0, N], with N equal to 100. The parameter *γ* was randomly selected from a uniform distribution from 0 to 1. We held the parameter *β* constant at a value of 2, and the cost (*c*) was set to 0.1. We investigated dynamic behavior across various initial cooperation levels, denoted as α, which ranged from 0 to 50%. Each experimental condition is denoted by {*α*, *c*, *β*}. Our primary focus during this observation was to track the evolution of cooperation levels, *CCC* values, and *γ* values.

**Figure 2:**
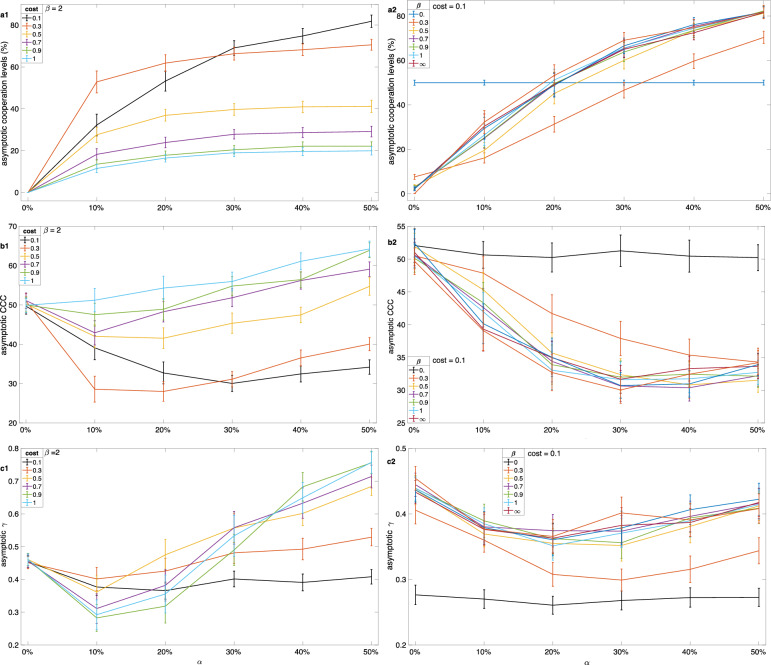
Asymptotic values of the population dynamics and the vertical bars represent standard deviations.

Figure 2 presents the asymptotic values of population dynamics, focusing on observing the behavior across different parameter ranges of {*α*, *c*, *β*}.

**Figure 3:**
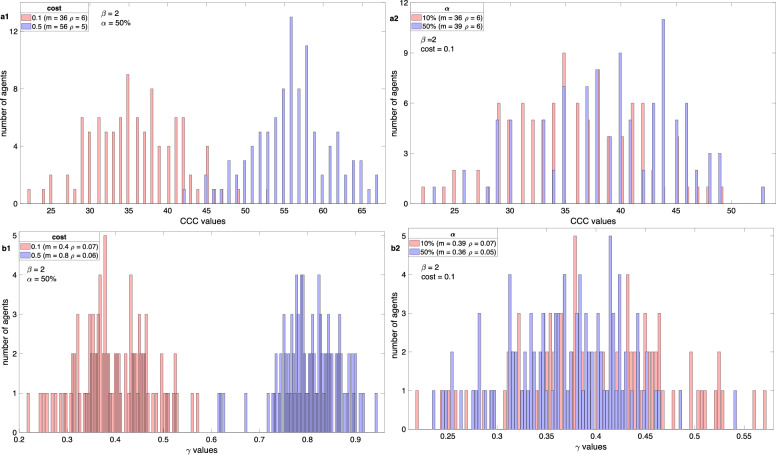
Distribution of *CCC* and *γ* values.

Figure 3 illustrates the distribution of *CCC* and *γ* values for specific parameter selections. The results illustrate insights into the distribution of conditional strategies and propensity-hold norms.

Figure 2 offers a comprehensive analysis of the evolutionary dynamics of cooperation and social norms. In Fig. 2a1, b1, and c1, we portray the asymptotic cooperation levels, *CCC*, and *γ*, respectively, as they vary with different cost values and initial cooperation levels while keeping *β* constant at 2. On the other hand, in Fig. 2a2, b2, and c2, we illustrate the asymptotic cooperation levels, *CCC*, and *γ*, respectively, as they vary with different *β* values and initial cooperation levels while maintaining a constant cost value of 0.1.

Specifically, Fig. 2a1 and b1 highlight that higher initial cooperation levels and lower contribution costs are conducive to the evolution of cooperation. Figure 2b1 emphasizes that initial cooperation levels are the driving force behind the dynamics for a fixed cost value, while *β* influences the dynamics except when *β* = 0. Meanwhile, Fig. 2c1 reveals that *γ* values tend to be higher when the initial cooperation is higher across various cost values. In Fig. 2c2, it becomes apparent that *β* does not exert a significant influence on the evolution of *γ*. In general, it is evident that lower contribution costs and higher initial cooperation levels create favorable conditions for the establishment of cooperation, and the parameter *β* plays a role in modulating dynamics except when *β* = 0, where agents resort to a coin toss decision.

In Fig. 3a1 and a2, we present the distribution of *CCC* values at the 20,000th generation while keeping *β* fixed at 2 and considering various cost levels (*c*). In Fig. 3a1, the initial cooperation level (α) is set at 50%, with a constant noise level. Conversely, Fig. 3a2 displays the distribution for different initial cooperation levels, with the cost fixed at 0. Moving on to Figs. 3b1 and b2, we observe the distribution of γ under similar experimental conditions.

Figure 3a1 and a2 reveal a distinct bifurcation in *CCC* values. When the contribution cost is lower (*c* = 0.1), the population adapts to lower *CCC* values, with a mean of 36. In contrast, with a higher cost (*c* = 0.5), the mean of the distribution increases to 56. Additionally, when the initial cooperation level is 50%, the population adapts to lower *CCC* values, averaging 39. Most of the *CCC* values in this scenario fall below 50. However, when the initial cooperation level is reduced to 10%, the mean *CCC* values remain higher, and the average value is 36, which is significantly lower than the initial cooperation level.

Regarding the distribution of *γ* in Fig. 3b1 and b2, it’s evident that there’s a noticeable bifurcation based on the contribution cost. When the cost is lower, the population tends to adapt to lower *γ* values, averaging around 0.4. Conversely, with higher contribution costs, the distribution tends to center around higher *γ* values, averaging approximately 0.8. Interestingly, there doesn’t appear to be a significant difference in the *γ* distribution concerning the initial cooperation levels. In both cases, the average values are close, with one averaging around 0.39 and the other at 0.36.

## Discussion

The study’s findings highlight the intriguing co-evolution of conditional cooperation and social norms among heterogeneous conditional cooperators. This co-evolutionary process hinges on agents making cooperative decisions based on the observed past cooperative behavior within their social group, coupled with free riders facing costs proportional to the current level of cooperation and the contribution cost. The research highlights the pivotal roles played by several factors: the initial cooperation level, the contribution cost, and the influence of noise parameters in accessing previous cooperation levels. These elements collectively determine the conditions necessary for the establishment of conditional cooperation. Notably, higher initial cooperation levels and lower contribution costs are critical factors in facilitating cooperation. Additionally, the noise factor modulates the coevolutionary dynamics across the parameters but does not thwart cooperation unless the noise factor level is high. Conversely, the study indicates that cooperative dynamics fail to materialize under unfavorable parameter combinations, including low initial cooperation levels or high contribution costs.

Let us discuss the role of these factors, such as initial cooperation (*α*), contribution cost (*c*), and the accuracy of social information about past cooperative actions in the population (*β*) on co-evolution of conditional cooperation and social norms.

The impact of initial cooperation levels has a dual effect, influencing both empirical and normative expectations. When a social group initially exhibits a high level of cooperation, it signals that cooperation is frequent, and free-riding may lead to sanctions. As a result, the population adapts to lower (*CCC*) values (Fig. 1b, Fig. 2b1, and 2b2), leading to increased cooperation levels, as depicted in Figs. 1 and 2a1. In such circumstances, the population tends to adapt to moderate values of the parameter *γ*. This adaptation reflects natural selection favoring individuals with moderate *γ* values, which protect them from experiencing high psychic costs when there are fewer cooperators in their environment. When individuals observe a scarcity of cooperators, they may not feel obligated to cooperate, potentially resulting in a lack of norm enforcement. Lower initial cooperation signals that cooperation is not a desired behavior in the population, and free riding is accepted. For instance, social distance is not followed when only a few individuals follow such rules in their vicinity^28,30^. The influence of empirical expectations has been observed in human societies^24,30^. Cooperation doesn’t establish itself when initial cooperation levels are low.

In situations characterized by high contribution costs, free riders can achieve greater rewards from public goods, and these increased payoffs can outweigh the costs associated with free riding. In these conditions, the agents within the population tend to adjust their strategies towards higher gamma values. This phenomenon can be attributed to the following factors: During the initial rounds, agents with lower gamma values who choose to free ride experience comparatively lower psychological costs than their counterparts with higher gamma values. Consequently, the agents with very high gamma values tend to become extinct within the population. However, as the population becomes increasingly homogeneous due to the proliferation of free riders, agents start imitating the strategies of their peers with a coin-toss-like approach. As a result, there is no further reduction in gamma values within the population, leading to an equilibrium where the average values settle at an intermediate level rather than being consistently low or high (see Figs. 2b2 and 3b1).

The influence of noise parameter *β* on the dynamics is moderate. When contribution costs remain manageable and initial cooperation levels are already high, even when social information is moderately accurate (with 0 < *β* < 2), cooperation will likely thrive. This can be observed in Fig. 2a2, where cooperation levels remain robust. In these favorable conditions, noise doesn’t exert a significant influence on the critical cooperation cost (*CCC*) and the parameter *γ*, as illustrated in Figs. 2a2, b2, and c2. Interestingly, the distribution of *γ* is sensitive to the contribution costs but not to the initial level of cooperation (see 2c2, 3b1, and 3b2).

Our model provides valuable insights into establishing social norms and conditional cooperation in the public goods games. It achieves this by connecting empirical and normative expectations to the social behavior of agents by highlighting their interactions with initial cooperation levels and contribution costs. However, certain potential limitations warrant consideration. The model does not encompass other parameters that can influence social behavior, including personal norms, and individuals might not be equally affected by their peers’ actions or conformity with expected peers’ behavior, as discussed elsewhere^106^. Moreover, the model’s concentration on a linear public goods game might not fully encapsulate the intricate dynamics of real-world social dilemmas ^107^. It is essential to acknowledge that social norms can also be internalized through top-down processes^24,41^, such as court rulings^34,46^ and institutional arrangements^2^. These mechanisms often involve role models, cultural leaders, and network structures, which are not explicitly incorporated into the model. It’s essential to recognize that a multifaceted interplay of individual behaviors, collective dynamics, cultural factors, and institutional structures influences the establishment and evolution of social norms^2,46,49^. The model becomes more intricate when dealing with larger population sizes, and it’s important to note that a high level of initial cooperation may not necessarily be conducive to establishing cooperation in such scenarios. This is because free riders often attain significantly higher payoffs in larger populations than in smaller ones.

In the real world, tracking and preventing free-riding behavior can be challenging, and enforcing punishments may not always be feasible. Norms tend to be most effective in tightly-knit communities where individuals can observe one another’s actions and enforce social sanctions. Additionally, in such close-knit communities, individuals are more likely to incur psychological or psychic costs for violating social norms, reinforcing cooperative behavior. This insight highlights the contextual nature of cooperation and the importance of social dynamics in shaping it within different settings.

In summary, the model comprehensively integrates crucial elements of social norms: The agents in the model consider empirical expectations or commonly observed social behavior when shaping their social preferences. Norm enforcement is not solely contingent on individual actions; it also hinges on the collective behavior of the majority within their social group. The results underscore that empirical expectations are pivotal in driving individual decision-making. At the same time, norm enforcement works to stabilize such behavior under favorable conditions, including higher initial cooperation levels, accurate social information, and lower contribution costs. The study’s findings illuminate the intricate interplay among individual behaviors, access to accurate social information regarding past group actions, and the repercussions of straying from established norms. This interplay significantly influences the development of cooperative norms within a societal framework. By comprehending these dynamics, we can glean valuable insights into the prerequisites for instating and fortifying cooperative behaviors. This, in turn, enhances our comprehension of social cooperation and the mechanisms underpinning normative systems.

### Supplementary Information

Supplementary Information.

## Data Availability

The datasets generated and/or analyzed during the current study are available in the [**Co-evolution_SN]** repository, https://osf.io/qxw3k/?view_only=5fd0335fdc0b4efe8aa3e0f802c3a88c**. DOI/accession number(s):** https://doi.org/10.17605/OSF.IO/QXW3K. I confirm that all methods were carried out in accordance with relevant guidelines and regulations. It is computer simulation data.
