# Supplementary material for: Co-evolution of conditional cooperation and social norm
Source: Sci Rep. 2023 Oct 3;13:16625. doi: 10.1038/s41598-023-43918-w (PMC10547722; doi:10.1038/s41598-023-43918-w)
Supplement: Supplementary file 1 — Supplementary Information. [file 41598_2023_43918_MOESM1_ESM.docx]

**Co-evolution of conditional cooperation and social norm**

Balaraju Battu

Computer Science, Science Division, New York University Abu Dhabi, UAE

Instructions for running the code and plotting

Data files can be found at: <https://osf.io/qxw3k/?view_only=5fd0335fdc0b4efe8aa3e0f802c3a88c>

DOI 10.17605/OSF.IO/QXW3K

All the following program files are kept in the same folder (co_evol_2023)

The function “ Conditional_SN.m” is a Matlab file implementation of the model.

The program file, “Conditional_SN_RUN.m” is for creating data using Conditional_SN.m function.

The program file, “randnlimt.m” is for creating mutations and is used by Conditional_SN.m.

The plotting program, “Conditional_SN_plotting.m” is available in the folder

co_evol_2023.
